# A novel integrated Markov-based model for vaccine cost-effectiveness in Lyme borreliosis and tick-borne encephalitis: implications for further research

**DOI:** 10.3389/fmed.2025.1676605

**Published:** 2026-01-21

**Authors:** Renata Šmit

**Affiliations:** University Medical Center Groningen (UMCG), Groningen, Netherlands

**Keywords:** Lyme borreliosis, tick borne encephalitis, prevention, modeling, cost effectiveness

## Introduction

The most common tick-borne diseases in Europe are tick-borne encephalitis (TBE) and Lyme borreliosis (LB). TBE is mainly prevalent in Central, Eastern, and Northern Europe, while LB is spread widely across Europe (1). Patients may be bitten by a tick carrying both pathogens, which could result in simultaneous infection with both diseases (2, 3).

While there are highly effective vaccines available for TBE, currently, there is no vaccine for LB. Additionally, there is no single combined vaccine that would protect against multiple tick-borne diseases, such as LB and TBE (4). Significant efforts are being made to develop a vaccine against LB and a single combined vaccine, which are potentially effective and cost-effective approaches to reducing the burden of these diseases (4).

In 2012, the first Markov model was developed to calculate the cost-effectiveness of TBE vaccination in Slovenia (5). This original model established a foundation for subsequent research and has supported some of the policy implications to date (6). Furthermore, based on the original article (5), Finland (7), Sweden (8–10), Estonia (11), and Germany (12) have performed their cost-effectiveness analyses on TBE vaccination. For the first time, the full burden of TBE, measured in Disability-Adjusted Life Years (DALYs), was estimated, including a correction factor of 4.5 to account for under-ascertainment and under-reporting (13).

Between 1999 and 2002, three studies (14–16) evaluated the cost-effectiveness of LB vaccination in the U.S, developing a decision tree (14) and later a Markov model (15, 16). The development of an anti-tick vaccine to prevent tick-borne diseases triggered (4) a study by Mihajlović et al., which assessed the cost-effectiveness of a potential single combined vaccine that would protect against both LB and TBE (17). The authors suggested a Markov model (17), informed by earlier research (5, 14–16). While the study attempts to evaluate a combined LB and TBE vaccine, some of the fundamental weaknesses significantly affect the reliability and relevance of its results (17).

This article presents several methodological, clinical, and economic weaknesses of the Mihajlović et al. model, and it introduces a *novel integrated Markov-based model* designed to address these weaknesses and guide future research in vaccine evaluation.

## Weaknesses of the study by Mihajlović J. et al. (2019)

### Independent modeling of LB and TBE without co-infection

In the study, the combined ICER is calculated, but it only includes independent LB and TBE and ignores co-infection. Co-infection occurs in 9.1%−16.7% of cases (3), and excluding it from the model would likely undervalue the vaccine's benefit. This could lead to an underestimation of cost-effectiveness.

### Lack of disease-specific ICERs

The study presents only the combined ICER. The authors did not calculate disease-specific ICERs and compare them with the combined ICER. This makes it difficult to determine the vaccine's true value.

### Tornado diagrams based on unreliable inputs

In Mihajlovic's study, the ranking in the tornado diagram is based on uncertain or speculative input parameters. This may lead to incorrect prioritization and misleading conclusions about the cost-effectiveness of the vaccine.

### Lack of probabilistic sensitivity analysis (PSA)

The authors state that a standard PSA was not performed due to “potentially imprecise results.” The absence of PSA means that the reader cannot assess uncertainty in relation to input parameter variability, which reduces the robustness of the cost-effectiveness results and the reliability of the findings. The lack of 95% confidence intervals further limits the credibility of the results.

### Lack of base-case and worst-case scenarios

The absence of base-case and worst-case scenarios makes it impossible to examine the range of cost-effectiveness outcomes under different input assumptions. This reduces the robustness of the model.

### Questionable incidence rate

The model used the incidence rate between 9/100,000 and 30.3/100,000 for LB and 1.51/100,000 to 3.53/100,000 for the TBE population. The authors did not specify for which year or period the incidence of TBE and LB was taken. From Table 1, it can be seen that the authors most recently used official data from the National Institute of Public Health of Slovenia (accessed on 20 April 2018). These rates used in the model are lower than historical values (e.g., 260/100,000 for LB and 10/100,000 for TBE) (18–20), which may lead to an underestimation of disease burden and distort ICER estimates.

**Table 1:** Summary of cost-effectiveness analyses of Lyme borreliosis (LB) and tick-borne encephalitis (TBE) vaccination.

**Study (Country, Year)**	**Disease**	**ICER**	**Perspective**	**Key assumptions (simplified)**	**Reference**
Mihajlović et al. (Slovenia, 2019)	LB + TBE	€46,061/QALY (two-dose, no boosters); €246,456/QALY (18-dose)	Societal	Two- or 18-dose schedule; no waning; low incidence; €50/dose	(17)
Šmit (Slovenia, 2012)	TBE	€15,128–€20,099/QALY (healthcare); cost-saving (societal)	Healthcare + societal	Three doses + boosters; lifetime protection; €20–24/dose + €2 admin; 70% exp.	(5)
Jürisson et al. (Estonia, 2015)	TBE	€24,576/QALY (50+); €60,572/QALY (all ages)	Healthcare	Three doses + boosters; €20–26/dose + €4 admin; no explicit waning	(11)
Shedrawy et al. (Sweden, 2018)	TBE	27,761 SEK/QALY (age 3); 99,527 SEK/QALY (age 40); 160,827 SEK/QALY (age 50)	Healthcare	Stockholm region; 3–4 doses + boosters; no explicit waning; 180–190 SEK/dose	(9)
Ollgren et al. (Finland, 2013)	TBE	€16,446/QALY (15/100,000); €120,081–€222,076/QALY (5/100,000)	Healthcare	Three doses only; no boosters; no waning; €54 for three doses + €5.3 admin	(7)
Müller et al. (Germany, 2024)	TBE	€82,358/QALY (60–85 years); €253,529/QALY (general population)	Healthcare	Three doses; no boosters; waning 5%/year after year 3; €50.12/dose	(12)
Shadick et al. (USA, 2001)	LB	$62,300/QALY (three doses); $72,700/QALY (three doses + boosters); $55,000/QALY (societal + boosters)	Healthcare + societal	Three doses ± yearly boosters; ~$150 series; productivity losses included	(15)

This table illustrates how cost-effectiveness estimates for TBE vaccination vary across countries due to differences in local epidemiology, vaccine schedules, and modeling assumptions. ICER values are reported as in the original studies. Cost-effectiveness thresholds differ by country. Vaccine schedules may include only the primary series or the primary series plus boosters. Incidence values reflect the epidemiological context assumed in each model. Currency values are not adjusted for inflation unless specified in the original publication.

LB, Lyme borreliosis; TBE, tick-borne encephalitis; ICER, incremental cost-effectiveness ratio; QALY, quality-adjusted life year; SEK, Swedish krona; USD, United States dollar.

### Misclassification of erythema migrans (EM) as a chronic sequela

EM is modelled as a potentially chronic condition lasting up to one year and leading to complications. This contradicts clinical guidance (21–23), which defines EM as a self-limiting early manifestation of LB that resolves within weeks to months following appropriate antibiotic treatment. Such misclassification can distort disease burden and misrepresent the benefit of the vaccine.

### Uniform utility weights for active LB and its sequelae

The model applies utility weights that do not differentiate between active LB and its sequelae in a cost-effectiveness analysis, overlooking differences in quality-of-life impact. This may inaccurately present the ICER estimates and the true burden of the disease.

### Unrealistic classification of TBE sequelae

Unlike other cost-effectiveness studies on modeling TBE for vaccination (5, 7–12), the study by Mihajlović et al. reports that mild sequelae develop only from mild cases of TBE, moderate sequelae only from moderate TBE cases, and severe sequelae only from severe TBE cases. This contradicts clinical evidence (24), which shows that moderate TBE can lead to severe sequelae or, for example, improve to mild sequelae (24). Such assumptions distort the course of disease and may lead to misleading results.

### Misalignment of the distribution of TBE sequelae

The model reports that 52.62% of mild TBE cases result in mild sequelae, 9.25% of moderate cases in moderate sequelae, and 50.26% of severe cases in severe sequelae. This implies that moderate TBE has the highest recovery rate (90.75%), followed by severe (49.74%) and mild (46.38%) cases. The authors did not provide any evidence to support their data. On the contrary, a study from Lithuania (24) reports that the highest recovery rate is for mild TBE and the lowest recovery is for severe TBE. Therefore, such a proportion of TBE sequelae as used in the Mihajlovic model (17) questions the model's validity.

## Unadjustable and misatributed cost of TBE sequelae

The model applies cost estimates for mild (€70), moderate (€122), and severe (€28,592) TBE sequelae, originally sourced from the 2012 study (5). In Mihajlovic's study, these values are used without adjustment. More importantly, Mihajlović et al. (17) cite these figures as 2019 estimates, despite their origin (5) in earlier literature. Recent studies by Müller et al. (12) and Scaggiante et al. (25) incorrectly refer to Mihajlović et al. as the primary source. This raises concerns regarding the reliability of ICER estimates and the validity of conclusions.

### Speculative vaccine parameters

The model is based on assumptions about the effectiveness, dosage requirements and cost of the single combined vaccine. Since these assumptions are speculative and uncertain, they can mislead stakeholders about the vaccine's true value. This might affect decisions on funding, development, and policy.

These weaknesses form the basis for the development of a novel model to support future cost-effectiveness evaluation.

## A novel integrated Markov-based model

To address several methodological and clinical weaknesses in Mihajlovi's study (17), this article introduces a novel integrated Markov-based model that improves the accuracy and relevance of the cost-effectiveness of vaccination for an LB and TBE. The model adheres to pharmacoeconomic guidelines (26, 27) and incorporates several key innovations that reflect real-world disease and clinical evidence. Unlike the previous model (17), the proposed model (28)

- represents both single and simultaneous infections of LB and TBE, enabling more accurate estimation of disease burden and vaccine impact,
- includes overlapping neurological symptoms between LB and TBE,
- models EM as a resolved condition, correcting Mihajlović et al.'s misrepresentation of EM as a chronic lesion,
- includes PTLDS as a potential outcome of disseminated or late-stage LB (e.g., neuroborreliosis, Lyme arthritis, acrodermatitis chronica atrophicans), in line with available clinical evidence.
- evaluates three vaccination strategies (LB-only, TBE-only, and combined) to provide a more comprehensive analysis for decision-makers,
- takes into account underreporting and under-ascertainment, improving the accuracy of incidence estimates and burden calculations,
- supports PSA and scenario modeling, enhancing the robustness of cost-effectiveness outcomes.

This proposed model (28) is also designed to work with real-world data and support its use in enhancing clinical relevance and reliability of the validation process. By improving the disease classification and modeling of LB and TBE, it supports a more reliable cost-effectiveness analysis and helps to assess the burden of disease more accurately. The originality of the model is shown in its structure and scope, and it can also be applied to other tick-borne diseases or diseases with multiple pathogens (28).

## Discussion

The integrated Markov-based model introduced in this study addresses key methodological and clinical weaknesses presented in the study by Mihajlović et al. (17). These weaknesses very likely distorted the ICER and reduced model relevance for decision-making. To address these weaknesses, the proposed integrated model incorporates co-infection, disease-specific ICERs and corrected clinical assumptions, making a more realistic and adaptable tool for evaluating the cost-effectiveness of vaccination against tick-borne diseases. It is also designed to apply real-world data, scenario modeling, and PSA, all important for health technology assessment (HTA) and regulatory decision-making. The development of a combined anti-tick vaccine targeting both LB and TBE further strengthens the need for such model flexibility. For example, Mihajlović et al. (17) calculated a cost-effectiveness threshold of €50,000/QALY gained in Slovenia. From a societal perspective, a single combined vaccine strategy with two doses is favorable, with an ICER of €46,061 per QALY gained for protection against both diseases (17). However, adults receiving 18 doses result in an increased ICER of €246,456 per QALY gained for combined protection, which far exceeds the acceptable threshold (17). This result shows that vaccine assumptions and epidemiological inputs can highly influence cost-effectiveness outcomes.

In contrast, other European studies (5, 7–12) have shown that TBE-only vaccinations are cost-effective, even with booster doses. In Slovenia, the ICER for TBE vaccination ranges from €15,128 to €20,099 per QALY gained from the healthcare payer's perspective (5). In Sweden, the ICERs vary depending on the age at which individuals are vaccinated (9). The ICERs are estimated at 27,761 SEK per QALY for those aged 3, 99,527 SEK per QALY for those aged 40, and 160,827 SEK per QALY for those aged 50 (9). Estonia reports an ICER of €60,572 per QALY for the general population and €24,576 per QALY gained for older adults (50+) (11). A Finnish study found TBE vaccination to be cost-saving at an incidence of 15 per 100,000, cost-effective at 10 per 100,000, but not cost-effective at five per 100,000 (7). Similarly, a German study reported ICERs of €253,529 per QALY for the general population and €82,358 per QALY for older adults when incidence rates were below five per 100,000 (12).

Most studies (5, 7–12) report that ICERs are cost-effective, especially when realistic dosing schedules and incidence rates are used. Mihajlović's ICER is higher despite applying a societal perspective, which favors vaccination (17). This suggests that the Mihajlovic model's structure and model of input may be responsible for the ICER being less cost-effective (17), rather than the value of the vaccine.

A study (15) on the cost-effectiveness of LB vaccination in the United States showed an estimated ICER of $62,300 per QALY gained for individuals who received three doses. When yearly boosters were included, the ICER increased to $72,700 per QALY gained. However, when productivity losses and boosters were incorporated, the ICER fell to $55,000 per QALY gained, indicating that societal perspectives may support LB vaccination more strongly than healthcare payer perspectives alone.

In their study, Mihajlović et al. (17) do not report a disease-specific ICER for LB and TBE and report EM as a chronic condition. This misrepresents the benefits of the vaccine and is likely to distort the estimated burden. In contrast, the integrated model introduced here addresses these weaknesses by distinguishing resolved EM from PTLDS. It also considers PTLDS to be a potential outcome of disseminated or late-stage Lyme borreliosis. Furthermore, it also uses disease-specific utility values to allow for more accurate estimation of QALYs and cost-effectiveness analysis.

Overall, these studies (5, 7–12, 14–17) demonstrate that the cost-effectiveness of vaccines varies depending on the pathogen, the population, the dosage and the economic perspective. The integrated model proposed here addresses these complexities and provides a tool for informing public health investment in tick-borne disease prevention. Using real-world data, it supports more realistic evaluation and aligns with HTA and regulatory needs.

## Conclusion

The study by Mihajlović (13) is an important step toward evaluating the cost-effectiveness of a single combined vaccine that targets LB and TBE. However, its full potential is limited by methodological weaknesses. To address these, this article proposes a novel integrated Markov-based model that is original in both structure and scope. The model can be adapted to multi-pathogen diseases. It is designed to enhance scientific standards and inform future decision-making.
